# Functional assessment of a constitutively activating *CASR* variant causing autosomal dominant hypocalcemia

**DOI:** 10.1210/jendso/bvag164

**Published:** 2026-08-10

**Authors:** Bryan K Ward, Bronwyn G A Stuckey, John P Walsh, Arthur D Conigrave, Suzanne J Brown, Scott G Wilson

**Affiliations:** Department of Endocrinology and Diabetes, Sir Charles Gairdner Hospital, Nedlands, WA 6009, Australia; Harry Perkins Institute of Medical Research, Centre for Medical Research, QEII Medical Centre, University of Western Australia, Nedlands, WA 6009, Australia; Department of Endocrinology and Diabetes, Sir Charles Gairdner Hospital, Nedlands, WA 6009, Australia; Keogh Institute for Medical Research, Nedlands, WA 6009, Australia; Medical School, University of Western Australia, Nedlands, WA 6009, Australia; Department of Endocrinology and Diabetes, Sir Charles Gairdner Hospital, Nedlands, WA 6009, Australia; Medical School, University of Western Australia, Nedlands, WA 6009, Australia; School of Life and Environmental Sciences, Charles Perkins Centre (D17), University of Sydney, Camperdown, NSW 2006, Australia; Department of Endocrinology, Royal Prince Alfred Hospital, Camperdown, NSW 2050, Australia; Department of Endocrinology and Diabetes, Sir Charles Gairdner Hospital, Nedlands, WA 6009, Australia; Department of Endocrinology and Diabetes, Sir Charles Gairdner Hospital, Nedlands, WA 6009, Australia; School of Biomedical Sciences, University of Western Australia, Nedlands, WA 6009, Australia; Department of Twin Research and Genetic Epidemiology, King's College London, London SE1 7EH, UK

**Keywords:** calcium sensing receptor, autosomal dominant hypocalcemia type 1, constitutive activation

## Abstract

**Context:**

A rare cause of hypocalcemia, autosomal dominant hypocalcemia type 1 (ADH1) arises from a gain-of-function variant of the calcium-sensing receptor gene (*CASR*).

**Objective:**

Three patients from 2 unrelated families, presenting with hypocalcemia and other biochemical parameters consistent with ADH1, were examined for variants in the *CASR* with the aim to functionally assess any variant detected to confirm the ADH1 diagnosis.

**Methods:**

Sanger sequencing of the coding region of the *CASR* from the 3 patients identified a single *CASR* variant that was generated by site-directed-mutagenesis in the *CASR* as a FLAG-tagged construct in the mammalian expression vector pcDNA3.1. The variant's expression in HEK293 cells (compared to FLAG-tagged wild-type [WT] receptor) was assessed by Western blot analysis and its activity measured following calcium dosing experiments using an IP-One enzyme-linked immunosorbent assay.

**Results:**

Sequence analysis revealed the presence of a heterozygous missense variant in the *CASR*, an adenine to guanine transition at nucleotide 1256 causing an asparagine to serine substitution at amino acid 419 (N419S) in the CaSR’s Venus flytrap domain in all 3 patients. The N419S variant showed a modest increase in expression compared to the WT receptor. Significantly, the IP-One assay demonstrated that the variant is constitutively active in the absence of Ca^++^ ions and that this gain-of-function is maintained at physiologically relevant Ca^++^ ion concentrations.

**Conclusion:**

The N419S *CASR* variant affecting 2 separate families is constitutively activating and therefore causative of ADH1. This is the first report of a constitutively active variant affecting the extracellular domain of the CaSR.

Hypocalcemia has many potential causes and while some may be obvious (eg, hypoparathyroidism arising post surgery), others may require rigorous and repeated biochemical testing [1, 2]. A rare cause of hypocalcemia, autosomal dominant hypocalcemia type 1 (ADH1), arises from gain-of-function mutations in the calcium-sensing receptor gene (*CASR*), which encodes the calcium-sensing receptor (CaSR), a G protein–coupled receptor crucial in maintaining calcium homeostasis [3-5]. The estimated prevalence of ADH1 is no greater than 1 per 25 000, although recent studies indicate that this disorder is considerably underdiagnosed [6, 7]. Less common still, autosomal dominant hypocalcemia type 2 (ADH2) is caused by gain-of-function mutations in the Gα_11_ protein that couples to the CaSR [8, 9]. By contrast, loss-of-function mutations of the *CASR* present in the heterozygous or homozygous state cause familial hypocalciuric hypercalcemia type 1 (FHH1) and neonatal severe hyperparathyroidism, respectively [9, 10]. Very rarely, neonatal hyperparathyroidism may arise in the heterozygous state where a dominant negative *CASR* variant is inherited from the father (or arises de novo) [11]. Loss-of-function mutations of the Gα_11_ protein or of the Arg15 residue of the adaptor-related protein complex 2 σ subunit cause FHH2 and FHH3, respectively [9]. ADH1 patients present with mild to moderate hypocalcemia in the context of inappropriately low to normal parathyroid hormone (PTH) levels; in some cases, elevated serum phosphate and/or hypomagnesemia may be a feature [12, 13]. Clinical symptoms occur in up to two-thirds of patients and may include muscle cramps/spasms, paresthesia, and in some cases, seizures [12, 13]. In addition, nephrocalcinosis sometimes occurs in ADH1 patients, predominantly following treatment with calcium supplements in association with activated forms of vitamin D. Basal ganglia calcification may be seen on imaging, and more serious complications including arrythmias, laryngospasm, and neuropsychological disorders occasionally occur [13, 14]. Considerable biochemical and clinical phenotype variation exists, even in family members heterozygous for the same mutation [15] with dietary intake of calcium and adequacy of vitamin D most likely playing a role in these differences. Asymptomatic cases of ADH1 are not treated; symptomatic patients may be administered calcium/vitamin D supplements with the aim of reducing symptoms by adjusting serum calcium levels to the lower end of the normal range to prevent marked hypercalciuria and nephrocalcinosis; in refractory cases recombinant human PTH or PTH analogues may be used, while calcilytics, negative allosteric modulators of the CaSR, are in phase 3 clinical trials [16, 17]. Certain activating variants give rise to a more severe form of ADH1 with Bartter syndrome [12, 13]. Well over 100 variants of the *CASR* associated with ADH1 have been reported with only a limited number being functionally characterized [18]. Most of the mutations are of the missense kind and predominate in the Venus flytrap (VFT) domain of the receptor, particularly in the homodimer interface of lobe 1 (residues 116-136) and in the 7-transmembrane (TM) domain unit, particularly within TM6-ECL3-TM7 (residues 818-845) [12, 18, 19]. Several deletion/nonsense mutations in the intracellular tail of the *CASR* have also been associated with ADH1 [12, 18]. The importance of functionally characterizing *CASR* variants has been highlighted previously, as in silico predictions regarding the pathogenicity of *CASR* variants cannot be relied on as being accurate, even when they appear to cosegregate with the disorder [12, 20, 21]. We present functional data on a previously uncharacterized *CASR* variant identified in 2 families with biochemically diagnosed hypocalcemia.

## Material and methods

### Patient biochemistry and genetic analysis

Patients attending the endocrinology clinic at Sir Charles Gairdner Hospital, Perth, for suspected calcium disorders underwent a full fasting metabolic study in which serum was analyzed for ionized calcium (adjusted to pH 7.4), and plasma for intact PTH, total calcium (corrected for albumin), and creatinine as described previously [20]. Serum 25-hydroxy-vitamin D was measured using an automated DiaSorin Liaison immunoassay analyzer (DiaSorin) and plasma phosphate analyzed as described previously [22]. Calcium and creatinine were measured on early-morning spot urine samples (second void) [20, 23]. All patients provided informed consent to biochemical and genetic testing that was performed as part of routine clinical care. Ethics approval for studies with humans involving the *CASR* was granted by the University of Western Australia Committee for Human Rights (project No. C04).

DNA for genetic analysis was extracted from leukocytes of heparinized blood samples according to standard procedures [24] or from EDTA-treated blood samples using the High Pure polymerase chain reaction (PCR) Template Preparation kit, version: 20 (Roche). Segments of the coding region that included intron-exon junction sites were amplified by PCR using primers and conditions as described previously [24]. Sanger sequencing of amplicons using BigDye terminator (version 3.1) chemistry was performed at the Australian Genome Research Facility. *CASR* variants were confirmed by repeat PCR and sequence analysis of the relevant amplicon. In silico analysis of the potential pathogenicity of the variant was performed using ANNOVAR software tools in conjunction with the University of Maryland Genetic Variation Interpretation Tool as a further guide to classification of the variant [21, 25].

### Mutagenesis, cell transfections, and Western blot analysis

The *CASR* missense variant was generated using a site-directed mutagenesis kit (Agilent Technologies) in wild-type (WT), C-terminally FLAG-tagged *CASR* cloned into the mammalian expression vector pcDNA3.1 [20, 23, 24]. Transfection quality DNA of empty vector, WT, and variant CaSR plasmids was prepared using the Pure Yield miniprep system (Promega Corp) and quantitated using a NanoDrop One spectrophotometer (Thermo Fisher Scientific). HEK293 cells (RRID: CVCL_0045, https://scicrunch.org/resolver/RRID:CVCL_0045) were propagated (in 25 cm^2^ flasks) in Dulbecco's Modified Eagles Medium (DMEM) containing 10% fetal bovine serum and antibiotics and 5 µg plasmid DNA (empty vector, WT, or variant) transfected into the cells (at ∼70% confluency) using lipofectamine 2000 (Life Technologies Invitrogen) prepared in antibiotic-free OPTIMEM medium (Invitrogen) as described previously [20]. Following 7 hours’ incubation at 37 °C in 5% CO_2_, antibiotic-free medium was replaced with DMEM propagation medium and reincubated. At 48 hours post transfection, cell monolayers were washed in phosphate-buffered saline and lysed in lysis buffer containing 100-mM iodoacetamide as described previously [20]. Protein was quantitated using the bicinchoninic acid protein assay kit (Pierce) with absorbance read at 560 nm on an iMark microplate absorbance reader (Bio-Rad Laboratories). For Western blot analysis, protein (60 µg) was separated under reducing conditions (∼200 mM dithiothreitol) on a sodium dodecyl sulfate polyacrylamide gel prepared using a TGX Stain-Free FastCast Acrylamide kit, 7.5% (Bio-Rad) and blotted on to nitrocellulose using a Trans-Blot Turbo Transfer System (Bio-Rad) set at standard SD protocol conditions. Membranes were probed with primary antibodies: either a mouse monoclonal antibody to FLAG-tag (RRID: AB_259529, https://scicrunch.org/resolver/AB_259529) to detect the CaSR or an α-tubulin antibody (RRID: AB_477593, https://scicrunch.org/resolver/AB_477593) as a protein-loading standard, followed by goat antimouse-horseradish peroxidase–conjugated secondary antibody (RRID: AB_258167, https://scicrunch.org/resolver/AB_258167).

All antibodies were from Sigma-Aldrich, with incubation conditions and wash buffers as described previously [20, 24]. Protein bands were visualized using Western Lightning Plus-ECL–enhanced luminescence substrate (Revvity Health Sciences) in a ChemiDoc XRS + Molecular Imager (Bio-Rad).

### IP-One assay

The IP-One competitive immunoassay, which measures accumulation of D-myo-inositol-1-phosphate following ligand activation of the CaSR, was performed as described previously [20] using the IP-One enzyme-linked immunosorbent assay (ELISA) kit (Cisbio Bioassays; catalog No. 72IP1PEA, RRID:AB_2904131, https://www.antibodyregistry.org/AB_2904131). In brief, WT or variant CaSR plasmid was transfected into HEK293 cells as described in the previous section and after an 8-hour incubation, cells were seeded into 24-well plates (4 × 10^5^ cells/well) and incubated for 48 hours. Following incubation, cells were first washed in Ca^++^-free DMEM, then in stimulation buffer (without calcium), prior to dosing with various concentrations of Ca^++^ ions (0, 1.5, 2.5, 4, 7, and 10 mM) in stimulation buffer for 1 hour at 37 °C in 5% CO_2_. Some wells were left untreated for standard processing for Western blot analysis performed as described in the previous section except that the amount of protein loaded was 40 µg rather than 60 µg. Dosed cells were lysed, and the lysate clarified by low-speed centrifugation and the supernatant transferred to the kit's 96-well ELISA plate for assay according to the manufacturer's protocol. Dosing experiments were performed in triplicate and IP-One assay values in duplicate with all assay points performed on the one plate. Absorbance values were measured on an iMark microplate absorbance reader (Bio-Rad) at 450 nm with a 620 nm correction (subtracted from the 450 nm values). A further correction was made for nonspecific binding and the duplicate assay values averaged.

### Statistical analysis

The percentage stimulation values were calculated by normalization to the WT receptor as follows: % stimulation = [1 − (absorbance_stim_/absorbance_nonstim_)], where absorbance_nonstim_ = the mean absorbance at 0 mM Ca^++^ ions for the WT receptor. The difference in mean percentage stimulation between variant and WT receptor at different Ca^++^ ion concentrations was assessed statistically via mixed-effects β regression, with run number treated as a random effect. Specific comparisons were made at 3 calcium concentrations (0, 1.5, and 2.5 mM). To adjust for multiple comparisons in the examination of differences between WT and N419S receptors, the statistical significance level α′ = 0.05/3 = 0.017 was used. All analyses were completed in the R statistical computing environment, version 4.5.1.

## Results

### Biochemical and clinical assessment of patients

Patient A was a 39-year-old woman with hypocalcemia detected on routine investigation during in-patient psychiatric assessment. She had a history of borderline personality disorder, posttraumatic stress disorder, and major depression with multiple previous psychiatric admissions. She complained of perioral paresthesia occurring most days, but no history of peripheral paresthesia, muscle weakness, cramps, or tetany. There was no family history of hypocalcemia. She was diagnosed with idiopathic hypoparathyroidism, with a differential diagnosis of ADH. Her symptoms responded to supplementation with calcium carbonate 600 mg daily.

Patient B1 was a 55-year-old man who presented with fatigue and muscle aches. He had a history of hypertension (treated with perindopril) and gout. His muscle aches responded to supplementation with calcium carbonate 600 mg daily. The differential diagnosis was idiopathic hypoparathyroidism vs ADH, and plasma calcium levels were requested from his 3 children. His 28-year-old daughter (patient B2) was found to be hypocalcemic and reported symptoms of muscle twitching of her lower limbs, fingers, and eyes, which responded to treatment with calcium carbonate 600 mg twice daily. Of his 2 sons, 1 was normocalcemic, and the other declined testing.

None of the patients had a history of neck surgery or irradiation, and none had clinical features of syndromes associated with hypoparathyroidism. None were treated with active forms of vitamin D.

All 3 patients exhibited ionized and total hypocalcemia with plasma PTH (inappropriately) within the reference range and urine calcium/creatinine ratios in the lower reference range (Table 1). Patient A exhibited hyperphosphatemia and patient B1 had mild vitamin D deficiency. None of the patients had hypomagnesemia (results not shown).

**Table 1:** Patient biochemical data

Patient	Sex	Serum ionized calcium (pH 7.4) (1.12-1.32 mmol/L)	Plasma total calcium (corrected) (2.15-2.55 mmol/L)	Plasma phosphate (0.8-1.50 mmol/L)	Urine calcium/creatinine ratio (M: 0.04-0.45 F: 0.10-0.58 mmol/mmol)	Serum 25-hydroxy-vitamin D (>50 nmol/L)	Plasma intact PTH (pmol/L) (RR below)
**A**	F	1.03	1.97	1.79	0.15	71	3.3*^a^*
**B1**	M	1.07	1.92	1.29	0.15	44	3.4*^b^*
**B2**	F	1.04	2.01	1.36	0.13	101	2.0*^c^*

Patients B1 and B2 are a father and daughter from the same family. Patient A is from a separate, unrelated family.

Abbreviations: F, female; M, male; PTH, parathyroid hormone.

Serum intact PTH reference ranges: *^a^*0.9 to 9.0 pmol/L, *^b^*0.7 to 7.0 pmol/L, *^c^*1.6 to 6.9 pmol/L.

### Genetic analysis of patients

Genetic analysis of each patient revealed a missense variant in the *CASR*, an adenine to guanine transition at nucleotide 1256 leading to an asparagine to serine substitution at amino acid 419 (NM_000388.4:c.1256A>G:p.(Asn419Ser) (N419S). This amino acid change occurs in the second part of lobe 1 of the VFT-sensing domain of the receptor [12, 18, 19]. While the amino acid corresponding to the variant is well conserved (GERP++ RS rank score 0.959), other bioinformatics data were ambiguous with respect to predicted pathogenicity, with SIFT, PolyPhen-2 HVAR, MetaLR, and FATHMM rank scores indicating that the variant is likely deleterious/damaging but combined annotation dependent depletion (CADD) Phred [25, 26] and Mutation Assessor rank scores suggesting a benign variant (Table 2). Without adequate phenotype/genotype cosegregation studies or convincing in vitro or in vivo functional assessment, application of the University of Maryland Genetic Variation Interpretation Tool classified the variant as a variant of unknown significance (VUS) satisfying evidence categories PM2, PP2 and PP3.

**Table 2:** Bioinformatics data for the Asn419Ser calcium-sensing receptor variant

Variant	SIFT score	PolyPhen-2 HVAR score	CADD Phred score	MetaLR rank score	Mutation Assessor rank score	FATHMM converted rank score	GERP++ RS rank score	SiPhy 29way logOdds rank score
**NM_000388.4:c.1256A>G:p.(Asn419Ser)**	Deleterious (0.042)	Probably damaging (0.992)	Likely benign (24.3)	Deleterious (0.715)	*^a^* Low (0.342)	Damaging (0.864)	Highly conserved (0.959)	Moderate to strongly conserved (0.758)

Combined annotation dependent depletion score cutoff for “likely deleterious” is greater than 30 [26].

Abbreviation: CADD, combined annotation dependent depletion.

^*a*^Signifies low deleterious probability.

### Western blot analysis

**Figure 1:**
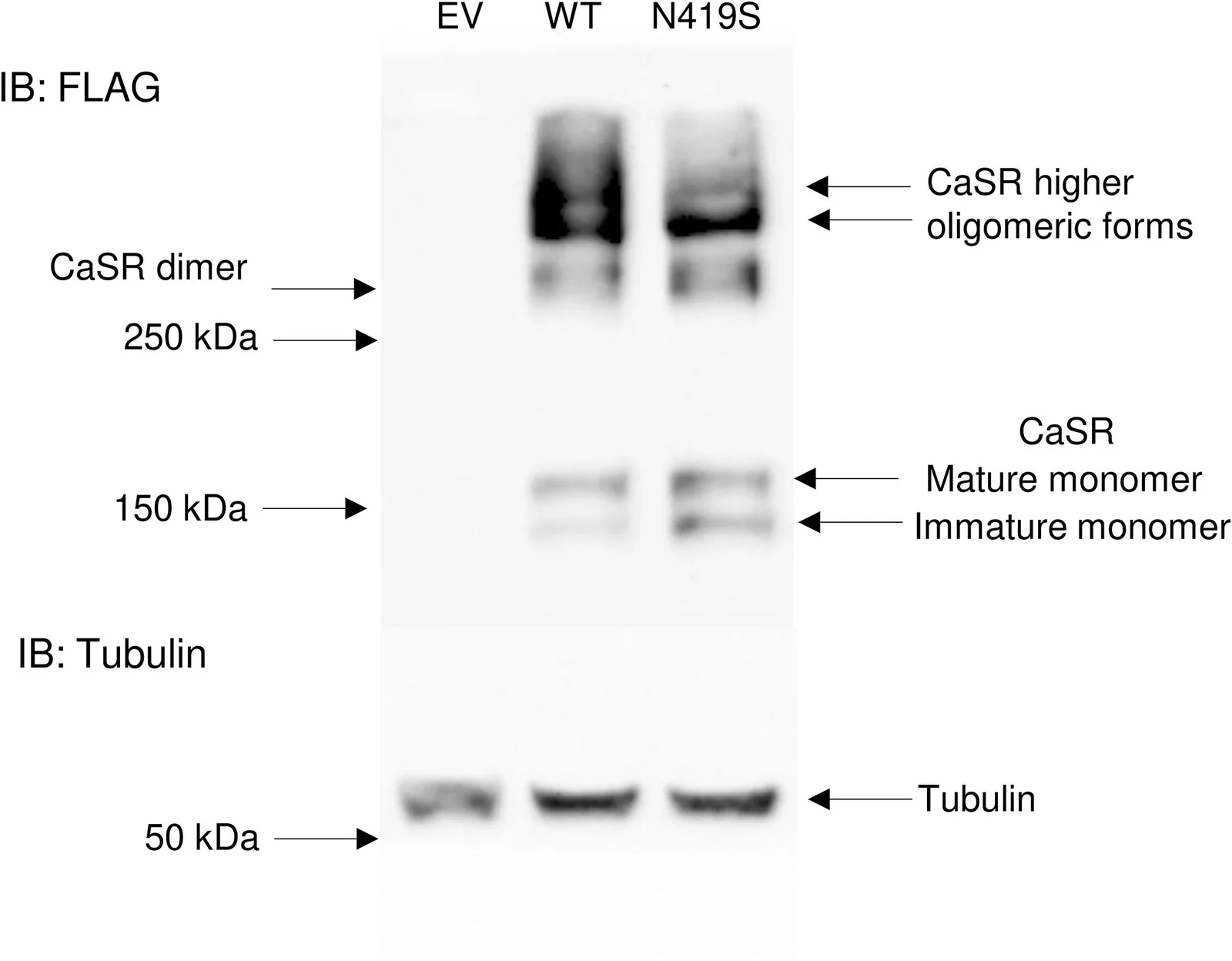
Western blot analysis comparing relative expression of FLAG-tagged wild-type (WT) and variant (N419S) CaSR. Empty vector (EV, untagged) was included as a background control. Plasmid DNA was transfected into HEK293 cells, and after 48 hours the cells were lysed and 60 µg protein (in reducing buffer containing urea and ∼200 mM dithiothreitol) separated on a 7.5% polyacrylamide gel, then blotted on to nitrocellulose and probed with anti-FLAG antibody to detect the CaSR and anti–α-tubulin antibody as a loading control. The blot is representative of 3 separate experiments.

**Figure 2:**
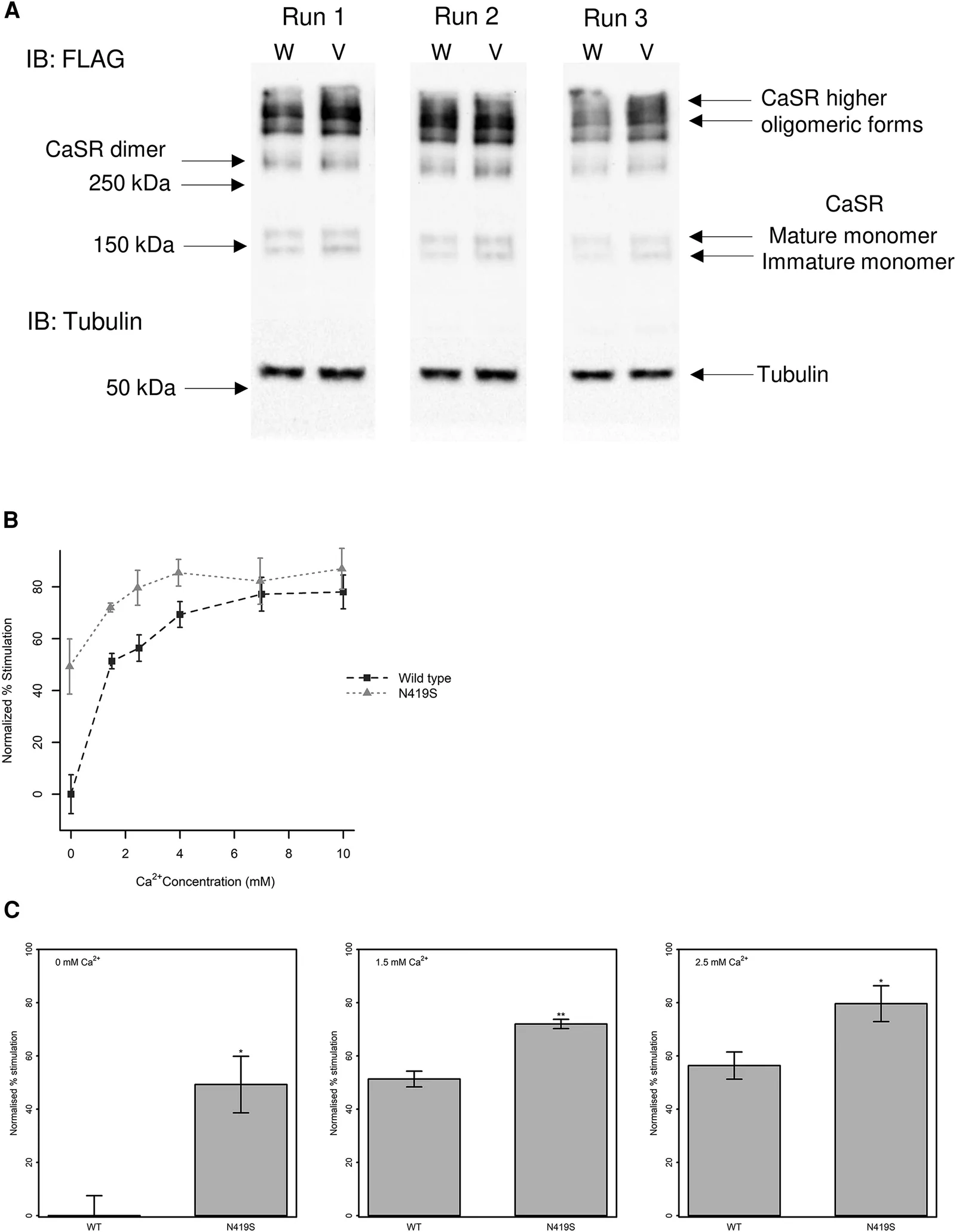
Comparative analysis of wild-type (WT) and variant (N419S) receptor expression and receptor activation following Ca^++^ stimulation using the IP-one enzyme-linked immunosorbent assay (with activation via the G_αq/11_-coupled phospholipase C cascade). A, Western blot analysis comparing the relative expression of WT and N419S variant (V) CaSR corresponding to each IP-One experimental run (Western blots were performed as described for Fig. 1 except that 40 µg of protein was used for separation). B, Dose response curves plotting mean percentage stimulation normalized to that of WT receptor (±2× SEM error bars) against Ca^++^ concentration for WT and N419S receptors for the 3 experimental runs. C, Bar plots comparing mean percentage stimulation (normalized to WT receptor) (±2× SEM error bars) for WT and N419S receptors at 0, 1.5, and 2.5 mM Ca^++^ concentrations for the 3 experimental runs (**P<.001; *P<.01).

Western blot analysis performed under reducing conditions comparing WT and N419S CaSR showed the expected monomeric forms of the receptor (immature 140 kDa and mature 160 kDa) as well as dimeric (∼300 kDa) and higher oligomeric forms in both cases (Fig. 1). The expression of the N419S variant, including the monomeric and dimeric forms, was slightly greater than that for the WT receptor and overall slight to moderate increased expression of the variant was a consistent finding in 2 additional experiments (not shown) and in 3 more performed in conjunction with the IP-One assay runs (Fig. 2A).

### Analysis of calcium-sensing receptor variant activation and reassessment of pathogenicity status

The N419S variant showed considerable activity (>50% of maximal) in the absence of added Ca^++^ ions when normalized to the WT receptor, and this increased activity also occurred at more physiologically relevant levels of Ca^++^ ions (1.5 and 2.5 mM); in fact, the activity of the N419S variant in the absence of calcium was similar to the activity of the WT receptor when stimulated with 1.5-mM calcium. However, the maximal activity achieved at higher, supraphysiological concentrations of Ca^++^ ions (7-10 mM), was similar for the variant and WT receptor (Fig. 2B). Mean normalized percentage stimulation values were significantly higher for the N419S variant compared to the WT receptor without added Ca^++^ ions (*P* < .01) and at physiological levels of Ca^++^ ions (1.5 and 2.5 mM; *P* < .001 and *P* < .01, respectively) (Fig. 2C). These results indicate that the receptor is constitutively active in the absence of calcium and that this activation is sustained at physiological levels of calcium. Based on these functional studies, reevaluation of the classification of this variant using the Genetic Variation Interpretation Tool designated it Likely Pathogenic (II), satisfying evidence categories PS3, PM2, PP2, and PP3.

## Discussion

In this study we examined 3 patients (2 from 1 family and 1 from an unrelated family) who presented with hypocalcemia accompanied by inappropriately low PTH. The biochemistry results were consistent with either idiopathic hypoparathyroidism or ADH. However, the family history of patients B1 and B2 suggesting autosomal dominant inheritance together with observations of only mild hypocalcemic symptoms in all 3 patients are suggestive of ADH. The findings that, in the absence of treatment with active forms of vitamin D, the urine calcium-creatinine ratios were inappropriately within the normal reference range is also suggestive of ADH. Genetic studies were initiated and evaluation of all 3 patients revealed a missense variant in *CASR* (NM_000388.4:c.1256A>G:p.(Asn419Ser) (N419S). It is worth noting that a son of patient B1 who was normocalcemic tested negative for the variant; however, extensive cosegregation studies were not undertaken. The variant is listed in ClinVar and has been previously linked to ADH [18, 27-29]. To our knowledge, however, N419S has not previously undergone functional characterization. Previous in silico analysis and bioinformatics assessment in this study classified the variant as a VUS. Our functional studies demonstrated that the variant receptor is constitutively active in the absence of added calcium when compared to the WT receptor, and that this gain of function is sustained at physiological Ca^++^ levels. Functional assessment permitted reclassification of the variant as Likely Pathogenic II using the Genetic Variation Interpretation Tool, confirming the diagnosis of ADH1 in these patients.

Roszko et al in 2022 [13] conducted a comprehensive review of ADH1 attributable to *CASR* variants in which 191 patients with sufficient clinical information were systematically analyzed. Overall, 27% of cases were asymptomatic physically, 32% of patients showed moderate clinical symptoms, and 41% displayed more severe symptoms. The patients in our study were either asymptomatic or only mildly symptomatic and were diagnosed in adulthood (age 28-55 years). The review by Roszko et al [13] reported that seizures were the most common clinical presentation. However, this presentation and the young median age of diagnosis (4 years) may reflect the susceptibility of the developing brain to a variety of metabolic insults including hypocalcemia [30]. Hypercalciuria and hyperphosphatemia were associated with more severe symptomatic cases [13]. Broadly, the severity of symptoms correlated with the level of hypocalcemia but apart from the genotypes causing the more severe Bartter cases, there was no definitive relationship between genotype and severity of symptoms, with siblings with the same genotype often exhibiting divergent severity or symptoms [13]. This agrees with the study by Sorheim et al [15], who examined a large ADH1 family in which considerable variation in calcium homeostasis of *CASR* variant (T151M) carriers was evident, ranging from normocalcemia to severe hypocalcemia with concomitant severe symptoms requiring treatment. Cerebral basal ganglia calcifications were common and occurred independently of vitamin D treatment, indicating that ADH1 per se predisposes to these calcifications. On the other hand, other studies indicate that there may be a genotype-phenotype correlation in ADH1. In addition to variants affecting TM6 (such as A843E) causing severe Bartter-type syndrome, variants in the TM5 domain may also predispose to a more severe phenotype [31, 32]. Finally, in the study by Sorheim et al [15], most asymptomatic/untreated family members demonstrated PTH levels at the lower end of the normal range, as occurred in our patients.

Typically, activating variants of the CaSR demonstrate a left shift in the Ca^++^ dose response curve compared with that for the WT receptor in cellular assays of signaling responses, such as phosphoinositide hydrolysis [33, 34]. We report here a constitutively activating *CASR* variant N419S located in the receptor's VFT (in the extracellular domain [ECD]) that exhibited more than 50% of maximal activity when normalized to the WT receptor under incubation conditions in which no Ca^++^ ions were added. Moreover, the variant is also significantly more highly activating at physiologically relevant concentrations of Ca^++^ ions when compared with the WT receptor. To our knowledge this is the first report of a constitutively activating variant localized to the ECD of the receptor. A constitutively active missense variant, A843E, located in the seventh TM domain, has been reported previously in the CaSR; in this case the constitutive activity was independent of the VFT domain in which all 4 Ca^++^ binding sites have been identified [35] and was not due to overexpression of the variant compared to the WT receptor [31]. Interestingly, although the A843E variant was activating compared to the WT at zero or physiological levels of Ca^++^ ions, it reached only 50% of the maximal activation achieved by the WT receptor at supraphysiological Ca^++^ levels [31]. By contrast, N419S in the present study exhibited a comparable maximal response to that observed for the WT receptor. In addition, the A843E variant is associated with a more severe clinical phenotype with predictably severe hypocalcemia together with Bartter syndrome in all affected patients [13, 31]; by contrast, the patients with N419S in the present study were either asymptomatic or only mildly symptomatic. This suggests that constitutive activity in vitro does not necessarily correlate with clinical disease severity.

The NM_000388.4:c.1256A>G:p.(Asn419Ser) variant found in our 3 patients was reported previously in a patient thought to have ADH1 [27], but functional studies were not undertaken and it has been reported in ClinVar as a VUS. The nucleotide variant replaces a codon for asparagine with a codon for serine, which are both polar amino acids with neutral (uncharged) side chains, with serine having a shorter side chain than asparagine. Both are capable of forming hydrogen bonds when exposed to the aqueous environment. The variant is not present in population databases such as gnomAD, indicating that it is not a common polymorphism. The N419S variant is located in the second part of lobe 1 of the receptor [19]. Interestingly, another ADH1 variant, D410E, located in this part of lobe 1, has been functionally characterized; however, unlike the N419S variant, the D410E variant is not constitutively active [36].

**Figure 3:**
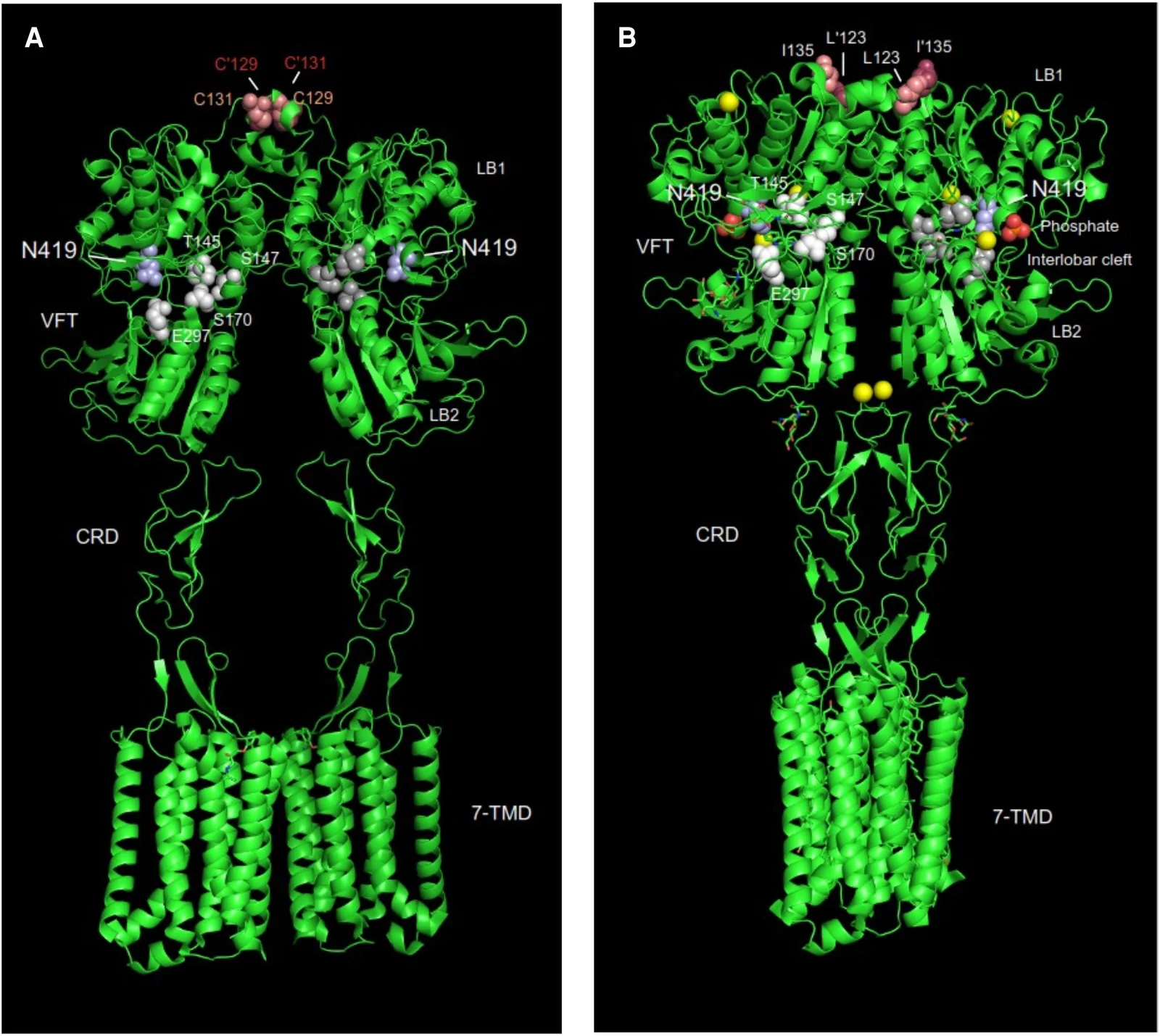
Location of N419 in A, inactive and B, active calcium-sensing receptor (CaSR) homodimers. The images were generated in Pymol using Protein Database Reference codes 7SIN (inactive) and 7SIM (active) for near-to-full length CaSR homodimers. Within the dimers the neighboring subunits are arranged antiparallel to one another. In the views selected, the interlobar cleft between ligand binding domains LB1 and LB2 faces toward the viewer in the right subunit and away from the viewer in the left subunit. Ca^2+^ ions are represented by yellow spheres and occupy up to 4 sites in the ECD of each subunit. Site 2 is positioned in LB1 above the interlobar cleft and is typically occupied in both inactive and active states. The other Ca^2+^ binding sites are occupied primarily in the active state. Residues (T145, S147, S170, E297) that contribute to the formation of the L-amino acid binding site in the interlobar cleft are represented in white (left subunit) or gray (right subunit). Cysteine residues, C129 and C131 in the left subunit, and C’129 and C’131 in the right subunit, form disulfide bridges that stabilize the dimeric structures (see [37]). They appear in the inactive (light red in the left and dark red in the right subunit) but not the active structures due to local disorder in the active subunits. The residues that are positioned at the ends of these disordered regions in each subunit of the active dimer (L123, I135, and L’123 and I’135) are shown. N419 (shown in light blue) is located in LB1 near 2 inhibitory phosphate-binding sites (see [35]). One phosphate molecule is shown in bright red. 7-TMD, 7-transmembrane domain; CRD, cysteine-rich domain; LB1, VFT ligand-binding lobe 1; LB2, VFT ligand-binding lobe 2; VFT, Venus flytrap domain.

While the gain-of-function activity of N419S might be explained in part by its mildly increased expression compared to the WT receptor, this would not account for its increased activity in the absence of added calcium ions. It is therefore likely that the variant induces structural changes that lead to its constitutive activity. Modeling showing the location of the N419 residue in relation to key determinants affecting activation of the CaSR is shown in Fig. 3. Ma et al [37] documented the importance of disulfide linkages between C131/C’131 and C129/C’129 in stabilizing the receptor in the inactive state; however, the N419S variant is distant from these cysteine residues and therefore would not be expected to lead to constitutive activity by disruption of these linkages (see Fig. 3). Geng et al [35] solved crystal structures of the CaSR both in inactive and active states and concluded that in addition to the integrity of several Ca^++^ binding sites, the integrity of an orthosteric tryptophan-binding site is critical for ECD closure leading to receptor activation. In addition, they found several anion-binding sites (most likely for inorganic phosphate) that were critical for maintaining either the inactive or active conformation (see Fig. 3); in particular in the so-called “anion-binding site No. 2,” the main chains of amino acid residues R415, I416, and S417, form hydrogen bonds with the side chains of R66, R69, W70, and S417 to hold 2 anions in place in the inactive state with only subtle changes leading to a single anion bound at this site in the active state. While N419 is not located in a critical binding site for Ca^++^ ions or activating L-AAs such as tryptophan [19, 35], it is close to residues R415, I416, and S417, which are critical for anion binding and to residues required for maintaining the structural integrity of the receptor for Ca^++^-dependent activation via R66, R69, and S417 [35]. Indeed, lobe-1 residue R66 is engaged in an H-bond across the interlobar cleft with lobe-2 residue S301 immediately proximate to S302 and S303, whose side chains support Ca^++^ binding site-3 [35]. Two possible explanations for the activating effect of N419S seem plausible: (i) that it disrupts hydrogen bonding networks, thereby impairing phosphate binding and thus stabilizing the activated receptor state; or (ii) that it disrupts an intrinsic inhibitory constraint on receptor activation that is dependent on the side chains of R66, R69, and S417 [35] and obviates the requirement for the H-bond network between S302 and S303 with water molecules associated with Ca^++^ bound in site 3 and between S303 and S417. For example, S419 could interact directly with S303 without the need for S417 or for Ca^++^ binding at S301 and S302. In this way, the receptor might adopt a partially active, intermediate state, even in the absence of Ca^++^, thereby explaining the current observation of constitutive receptor activation.

In conclusion, we identified a *CASR* variant (NM_000388.4:c.1256A>G:p.(Asn419Ser) in 2 families whose members displayed biochemical parameters consistent with ADH1. Functional characterization of the variant demonstrated that it exhibits constitutive activity in the absence of added Ca^++^ ions and that this gain of function was sustained at physiological calcium concentrations. The variant occurs in lobe 1 of the VFT domain and is the first report of a constitutively active variant occurring in the ECD of the CaSR. Our results support the identification of N419S as a causative variant for ADH1.

## Data Availability

Some or all of the datasets generated and/or analyzed during the current study are not publicly available but are available from the corresponding author by request.

## Abbreviations

ADH1: autosomal dominant hypocalcemia type 1
*CASR*: calcium-sensing receptor gene
CaSR: calcium-sensing receptor (protein)
DMEM: Dulbecco’s modified eagle medium
ECD: extracellular domain
ELISA: enzyme-linked immunosorbent assay
FHH1: familial hypocalciuric hypercalcemia type 1
HEK293: human embryonic kidney 293 (cell line)
IP-One: D-myo-inositol 1 phosphate
LB1/LB2: ligand-binding lobes 1 or 2
Opti-MEM: optimal (reduced serum) minimal essential medium
PCR: polymerase chain reaction
PTH: parathyroid hormone
SEM: standard error of the mean
TM: transmembrane
VFT: Venus flytrap (domain)
VUS: variant of unknown significance
WT: wild-type
